# Neurophysiological and Psychometric Outcomes in Minimal Consciousness State after Advanced Audio–Video Emotional Stimulation: A Retrospective Study

**DOI:** 10.3390/brainsci13121619

**Published:** 2023-11-22

**Authors:** Rosaria De Luca, Paola Lauria, Mirjam Bonanno, Francesco Corallo, Carmela Rifici, Milva Veronica Castorina, Simona Trifirò, Antonio Gangemi, Carmela Lombardo, Angelo Quartarone, Maria Cristina De Cola, Rocco Salvatore Calabrò

**Affiliations:** IRCCS Centro Neurolesi Bonino Pulejo, 98124 Messina, Italy; rosaria.deluca@irccsme.it (R.D.L.); paola.lauria@irccsme.it (P.L.); francesco.corallo@irccsme.it (F.C.); carmela.rifici@irccsme.it (C.R.); milva.castorina@irccsme.it (M.V.C.); simona.trifiro@irccsme.it (S.T.); antonio.gangemi@irccsme.it (A.G.); carmela.lombardo@irccsme.it (C.L.); angelo.quartarone@irccsme.it (A.Q.); mariacristina.decola@irccsme.it (M.C.D.C.); roccos.calabro@irccsme.it (R.S.C.)

**Keywords:** minimally conscious state, acquired brain injury, neurorehabilitation, multi-sensory stimulation

## Abstract

In the last ten years, technological innovations have led to the development of new, advanced sensory stimulation (SS) tools, such as PC-based rehabilitative programs or virtual reality training. These are meant to stimulate residual cognitive abilities and, at the same time, assess cognition and awareness, also in patients with a minimally conscious state (MCS). Our purpose was to evaluate the clinical and neurophysiological effects of multi-sensory and emotional stimulation provided by Neurowave in patients with MCS, as compared to a conventional SS treatment. The psychological status of their caregivers was also monitored. In this retrospective study, we have included forty-two MCS patients and their caregivers. Each MCS subject was included in either the control group (CG), receiving a conventional SS, or the experimental group (EG), who was submitted to the experimental training with the Neurowave. They were assessed before (T0) and after the training (T1) through a specific clinical battery, including both motor and cognitive outcomes. Moreover, in the EG, we also monitored the brain electrophysiological activity (EEG and P300). In both study groups (EG and CG), the psychological caregiver’s aspects, including anxiety levels, were measured using the Zung Self-Rating Anxiety Scale (SAS). The intra-group analysis (T0-T1) of the EG showed statistical significances in all patients’ outcome measures, while in the CG, we found statistical significances in consciousness and awareness outcomes. The inter-group analysis between the EG and the CG showed no statistical differences, except for global communication skills. In conclusion, the multi-sensory stimulation approach through Neurowave was found to be an innovative rehabilitation treatment, also allowing the registration of brain activity during treatment.

## 1. Introduction

Disorders of consciousness (DoC) refer to clinical conditions characterized by a lack of consciousness, mainly caused by heart failure, traumatic brain injury (TBI), hemorrhagic and ischemic stroke [1,2,3,4]. After a severe acquired brain injury, patients often manifest long-term alterations in their level of consciousness. Briefly, the coma state is a condition that develops after a head injury or from a temporary loss of oxygen to the brain, in which the patient has no residual awareness. It is possible to recover nearly completely from this state or to progress to a DoC, such as a vegetative state (VS) (more recently named unresponsive wakefulness syndrome, UWS), in which the patients recover the sleep–wake cycle, without awareness/consciousness of themselves or the surrounding environment. In the case that patients regain partial awareness, they are diagnosed as affected by a minimally conscious state (MCS) [5]. MCS individuals have been recently subcategorized according to the complexity of patients’ behaviors: MCS+ presents high-level behavioral responses (i.e., command execution, understandable verbalizations, or non-functional communication), whereas MCS- has low-level behavioral responses (i.e., visual pursuit, localization of noxious stimulation or contingent behavior such as appropriate smiling or sobbing in response to emotional cues) [6]. The diagnosis of DoC is based on clinical findings during the neurologic examination, including the elicitation of brainstem reflexes and the observation of spontaneous motor behavior and reactions to environmental stimuli. It is noteworthy that behavioral observations should be performed in an optimal environment and in the absence of sedation medications. In addition, clinicians can administer different standardized rating scales to achieve a correct diagnosis. Among these, the most used is Coma Recovery Rating Scale-Revised, (CRS-R) consisting of six subscales designed to assess auditory function, receptive and expressive language, visuo-perception, communication ability, motor functions, and arousal level. The Sensory Stimulation Assessment Measure (SSAM), Wessex Head Injury Matrix (WHIM), Western Neuro Sensory Stimulation Profile (WNSSP), Sensory Modality Assessment Technique (SMART), and Disorders of Consciousness Scale (DOCS) are also used in the patients’ differential diagnosis [7]. On the other hand, Glasgow Coma scale should not be considered for the bedside behavioral evaluation, due to its lack of validity and standardization [7], and it is more appropriate for patients in the acute phase of the brain injury. 

Since differential diagnosis has a pivotal role for the clinical management of these patients, the administration of clinical rating scales alone is not enough to reach the right diagnosis [8]. Neurophysiological measures, like electroencephalogram (EEG), can provide helpful information regarding diagnosis and recovery in DoC patients. According to a recent study [9], EEG biomarkers are considered feasible and accurate for the diagnosis of DoC. Indeed, these biomarkers may give important information regarding recovery prediction and prognosis classification of DoC patients [9]. Combining methodologies (i.e., behavioral and neurophysiologic tools) could be a promising strategy to achieve diagnostic accuracy and prognostic specificity in these patients [10,11]. 

Another important issue of DoC management is the therapeutic alliance with their caregivers, who are often their family members. In fact, active participation in the care process can help caregivers to avoid feelings of abandonment and frustration [12,13,14]. Among the available rehabilitation interventions [15,16,17,18,19,20,21,22] for DoC patients, sensory stimulation (SS) refers to those approaches aimed at promoting arousal and behavioral responsiveness by the application of environmental stimuli [23]. SS includes the presentation of different stimuli which are simple, frequent and repetitive, possibly autobiographical and with emotional content. During a SS session, the stimuli can be administered through different sensory channels (e.g., auditory, visual, tactile and olfactive) with a moderate-to-high intensity [24]. In addition, SS is a minimally invasive, non-dangerous, cheap, and simple to apply methodology in the rehabilitation field [25,26]. Nowadays, SS can be provided with innovative rehabilitation systems, like the Neurowave (Khymeia, Padova, Italy) [27]. This tool allows multisensory stimulation as well as the registration of brain activity through the P300, by using an EEG cuff. Despite the potential role of Neurowave in inducing advanced stimulation in DoC, there is little evidence [28,29] on this important tool for clinical practice. 

For these reasons, the primary aim of this retrospective study was to evaluate clinical and neurophysiological effects of multi-sensory and emotional stimulation provided by Neurowave in MCS patients compared to a conventional SS treatment. Our secondary aim was to monitor the psychological status of MCS’s caregivers as well as the satisfaction of caregivers via experimental training with Neurowave. 

## 2. Materials and Methods

### 2.1. Study Design and Population

Forty-two patients (mean age 51.8 ± 15.32) affected by MCS and their caregivers who attended the Semi-Intensive Care Unit of the IRCCS Centro Neurolesi “Bonino-Pulejo” (Messina, Italy), from May 2020 to June 2022, were included in our analysis using an electronic recovery system data (Table 1). 

This retrospective study was conducted in accordance with the 1964 Helsinki Declaration and approved by IRCCS Centro Neurolesi Bonino-Pulejo Research Institute Ethics Committee (ID: IRCCSME-20-2023, 19 April 2023). 

Through the retrospective nature of the study and using electronic medical records for extraction purposes, the scoring bias was minimized. We used motor and cognitive parameters to select the appropriate MCS patients who had been treated with Neurowave. The use of this innovative tool was part of the normal training of some patients attending our unit. The clinical evaluations, which we collected retrospectively, were carried out at the beginning and at the end of the training by the rehabilitation team (neurologist, physiatrist, nurse, physiotherapist, psychologist). 

Inclusion criteria were: (1) diagnosis of MCS following an acquired brain injury (vascular or traumatic), according to clinical and neuroradiological findings; (2) age range between 18 and 70 years; and (3) the presence of a caregiver. 

Patients having received multiple rehabilitation cycles as well as those with deafness and/or blindness were excluded.

All patients’ caregivers gave their written informed consent to participate in the study and data publication. We also asked the caregivers for their consent to publish the study results.

### 2.2. Procedures

The included patients were equally divided into two groups, having the same demographic and medical characteristics but different rehabilitation treatments. The experimental group (EG) received a multisensorial stimulation with an advanced audio–video stimulation tool focused on emotional and autobiographical stimuli using the Neurowave system, whereas the control group (CG) received a conventional multisensory stimulation without the innovative system. The rehabilitation protocol for both groups consisted of one hour a day training, three times a week, for 24 consecutive weeks, as per clinical practice in our rehabilitation unit. Each MCS patient was evaluated by the rehabilitative team (neurologist, psychiatric therapist, physiotherapist, and speech therapist) through the administration of the clinical scales and the neuropsychological battery before (T0) and after (T1) the treatment. Furthermore, in the EG, the EEG signal P300 was recorded in a resting state in the same day of the clinical assessment as well as at the end of the advanced neurorehabilitation cycle with the Neurowave. In fact, after the last experimental training (T1), the patient was provided with the same clinical and psychometric battery as designated at baseline, and was submitted to an EEG recording in resting state. All MCS patients were evaluated using a multidimensional screening tool, reported in Table 2, including motor (i.e., weakness, altered tone, balance and incoordination) and cognitive level/function [30,31,32,33,34,35]. 

**Table 2:** Description of psychometric and clinical outcomes administered for the assessment of MCS patients.

Scale/Test	Domain	Short Description
The Functional Independence Measure (FIM)	Motor and Cognitive Functioning	FIM, an 18-item (13 motor (motFIM) and five cognitive (cognFIM)) measurement tool, which explores an individual’s physical, psychological and social function, was used to determine the level of dependence of patients in daily life. This tool is used to assess a patient’s level of disability as well as the change in patient status in response to rehabilitation or medical intervention [30].
The Trunk Control Test (TCT)	Motor Impairment	TCT is used to evaluate motor impairment, and it correlates with eventual walking ability, as it tests rolling to each side, maintaining balance in the sitting position and sitting up from lying down [31].
The Global Communication Scale (GC)	Communication Abilities	GC, a specialist language questionnaire of verbal and non-verbal abilities, was used to investigate global communication. Response options range from 0 to 22 [32].
The Levels of Cognitive Functioning (LCF)	Cognitive Functioning	LCF is one of the earlier developed behavioral scales used to assess cognitive functioning in post-coma patients. It systematically describes and categorizes a patient’s level of consciousness and cognitive and behavioral functioning through which the patient typically progresses [33].
The Simplified Evaluation of CONsciousness Disorders (SECONDs)	Level of Consciousness	SECONDs is a tool composed of 8 items: arousal, localization to pain, visual fixation, visual pursuit, oriented behaviors, command-following, and communication (both intentional and functional). It is a short behavioral tool developed to diagnose brain-injured patients in time-constrained settings. This scale examines command-following, communication, visual pursuit, fixation, pain localization, oriented movements and arousal [34]. Aubinet et al. (2021) showed that SECONDs, compared to CRS-R, requires less examiner training, and the resulting score is directly related to a level of consciousness, ranging from EMCS (8), MCS+ (6–7), MCS− (2–5), UWS (1), to the coma (0) [35].

Moreover, the Zung Self-Rating Anxiety Scale (SAS) was administered to the caregivers of the enrolled patients. It is a 20-item self-reported assessment **tool** built to measure anxiety levels, and it is based on investigating cognitive, autonomic, motor and central nervous system symptoms to better assess the psychological caregiver’s functioning [36]. Each question is scored on a Likert-type scale of 1–4 (based on these replies: ‘a little of the time’, ‘some of the time’, ‘a good part of the time’, ‘most of the time’). Some questions are negatively worded to avoid the problem of a set response. 

Lastly, we evaluated the globally perceived quality of the Neurowave integration in the current clinical practice by a structured interview and a questionnaire with multiple answers designed by the rehabilitation team, with a focus on specific items: (1) team participation; (2) skills and reliability of the staff; (3) usefulness of the service in the emotional management of family members’ pathology; and (4) whether the caregiver would recommend the use of the Neurowave,

### 2.3. Neurowave and ERPs Parameter Recording 

MCS patients in the EG were stimulated with the Neurowave system (Neurowave, Khymeia s.r.l, Padua, Italy) with simultaneous acquisition of Evocated Related Potentials (ERPs), i.e., the P300, three times/week, for 24 consecutive weeks. 

**Figure 1:**
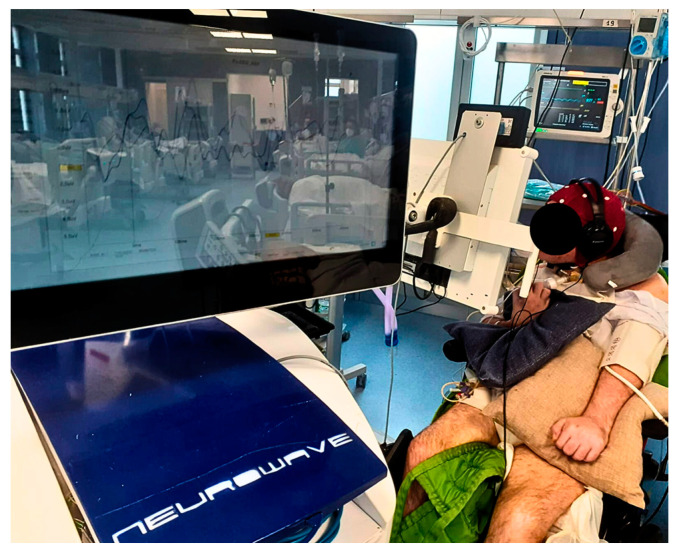
This figure shows a MCS patient during Neurowave training with the concomitant P300 registration. Legend: The picture was taken with the consent of legal guardian of the MCS patient, and it shows the patient watching the screen during the multisensory and emotional stimulation with the Neurowave and the concurrent registration of P300.

The Neurowave is an innovative and technologically advanced device that allows the programming and automated administration of multi-sensory stimulation, including images, movies, and sounds, as well as patient-specific memories. Indeed, its technology is particularly suitable for the field of severe DoC according to the patient’s disability level. Neurowave has excellent ergonomic characteristics for therapists, since it requires minimum space, and it can be easily moved from one bed to another. In addition, the Neurowave is equipped with a simultaneous system for the recording of multiple biophysiological signals, including the P300. The MCS patients underwent P300 recording during the first and last Neurowave sessions (see Figure 1). The Neurowave training was implemented in the current clinical practice.

ERPs were detected from 3 Ag/AgCl electrodes placed above the midline of the scalp (Fz, Cz, Pz,), according to the International Ten-Twenty System methodological set up for linking earlobes with a forehead ground [37,38,39]. Electro-oculograms were verified with four electrodes placed lateral to the outer canthus and above and below the left eye. Data were digitized at a sampling rate of 256 Hz and filtered with a band-pass 0.15 and 30 Hz. A notch filter was used. 

We have administered an intensive and repetitive task-oriented sensory-motor stimulation, using audio–video personalized materials with emotional significant for each patient, such as five visual and five audio stimuli target, including personal images, personal events of life, family members and partners or friends, pets and similar issues, as well as audio familiar/autobiographical registrations or individual sounds (work, home, hobbies etc). Stimuli had a duration of 500 ms for standard and target image. The interval inter-stimulus was set up to 800 ms. The occurrence of the rare stimulus has been set up to 20% and the appearance of the images was set up as ‘random’ modality. These audio–video stimulations were divided into sessions lasting 60 min [26,40]. 

### 2.4. Conventional Multisensory Stimulation 

Patients in the CG received conventional sensory stimulation (CSS). This approach is defined as multimodal because it usually involves the stimulation of different sensory modalities. Multi-sensory therapy is an activity which usually takes place in a dedicated environment, i.e., a sensory room, where patients experience a range of un-patterned visual and auditory stimuli, administered face to face with therapist or caregiver, using a traditional paper and pencil approach. 

Before starting each multisensory training, (advanced and not) the caregiver (which was included as a “co-therapist” in the multidisciplinary team) was constantly supported by rehabilitative methods, compiling the biographical format during a semi-structured interview focused on five main aspects of the DOC patient’s life. CSS was realized in a dedicate room, where the therapist used a personalized materials and information during each CSS’s session, characterized by: (i)autobiographic experience (work activity/tasks; people of emotional support; main events of patient’s life; the most significant places); (ii) personal identity(professional/domestic skills; lifestyle habits/sports activity; interests or hobbies; self-care habits; eating habits/tastes and/or favorite dishes; meaningful travels; (iii) individual context (favorite objects; odors/fragrances/perfumes/essences/preferential or habitual used; favorite colors, music and songs; family voices, familiar registrations; photo/registration of their pets; significant environments/spaces/places; (iv) photographs or videos with familiar figures, such as parents, children, friends; and (v)relevant emotional events (transfers; mourning; emotional traumatic events). During the sensory training session, the psychiatric technician and the psychologist administered these different kinds of audio–visual stimuli (e.g., colorful images, musical videos, and material contents), characterized by both neutral and personalized stimuli for the patients. Each conventional multisensory session lasted about 60 min, but the time can be reduced, in relation to the patient’s degree fatigue and the stability of vital parameters, monitored by the nursing staff. 

#### Statistical Analysis

Continuous variables, including age of both patients and caregivers, were expressed as mean and standard deviation, whereas psychometric (e.g., LFC, SECONDs, TCT, FIM, GC and SAS) and neurophysiological (e.g., P300 values) outcomes were expressed as median and first–third quartile, as appropriate. Categorical variables (e.g., gender, education and aetiology of MCS, degree of kinship) of both patients and caregivers were expressed as frequencies and percentages. 

According to the Shapiro–Wilk normality test for each variable, we performed a non-parametric analysis. Thus, linear correlations between variables were calculated by using the non-parametric Spearman rank correlation coefficient, between-group differences at baseline by the Mann–Whitney U test, and within-group changes in psychometric values and P300, i.e., between pre- (T0) and post- (T1) treatment within the same group, through the two-tailed Wilcoxon signed rank test.

Using the car package of R, for any outcome measure, an analysis of covariance (ANCOVA) was performed. The model had the test score at T1 as the dependent variable, the categorical variable ‘Group’ (1 = experimental; 0 = control) as the independent variable, and the outcome score at baseline (T0) as covariate. We also performed ANOVA to verify whether the model was significantly different when we fitted it, including the interaction term effect “outcome score at baseline x categorical variable”. 

Statistical significance was set at a bilateral α level of 0.05. All the analysis were conducted on the open-source software R 4.1.3 (Vienna, Austria) for Windows [41].

## 3. Results

All enrolled subjects completed the training without reporting any adverse events. No significant differences were found at baseline (T0) between the two groups regarding age, gender, education, aetiology, degree of kinship and psychometric/outcome scores (see Table 1). 

In the EG, the within-group analysis (T0–T1) showed statistical significances in all patients’ outcome measures, including FIM (*p* < 0.004), LCF (*p* < 0.005), SECONDs (*p* < 0.0002) TCT (*p* < 0.007), GC (*p* < 0.001). The caregivers showed a statistical significance in depression status measured with SAS (*p* < 0.001) (see Table 3). 

**Figure 2:**
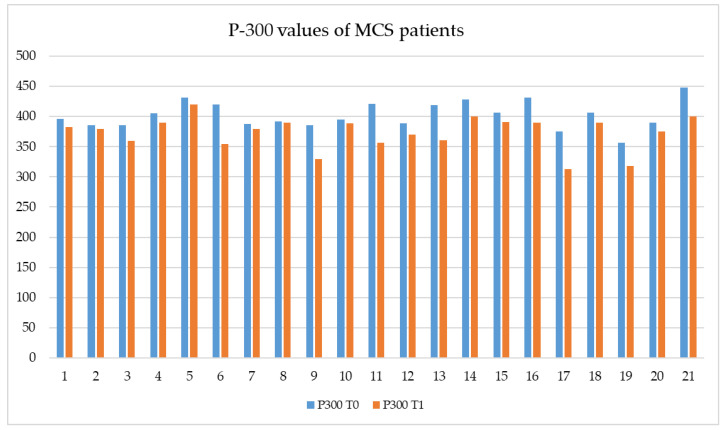
The histogram shows P300 values (*X*-axes) of all subjects (*Y*-axes) in the experimental group, before (T0) and after multisensory stimulation (T1) using Neurowave. Legend: The graphic shows P300 values (vertical or *X*-axes) of each MCS patient (horizontal or *Y*-axes) in the EG, before (in blue) and after (in orange) the multisensory emotional stimulation with the Neurowave.

Additionally, in the EG, we found a statistically significant improvement of the P300 features (*p* < 0.001) after the training (see Figure 2). 

In the CG, the within-group analysis showed statistical significances only in the LCF (*p* < 0.01), SECONDs (*p* < 0.005) and GC (*p* < 0.02), whereas caregivers showed the same statistical significance in SAS (*p* < 0.001) as the EG.

The ANCOVA analysis showed significant differences between treatment effects only in SECONDs (*p* < 0.001) and GC (*p* = 0.01), as reported in Table 4. Potential trends are also visible in FIM and SAS scores, although they did not reach statistical significance. In these two scales, we found a significance of the interaction term “outcome score at baseline x categorical variable” in ANOVA results: FIM (F = 8.887, *p* = 0.005) and SAS (F = 10.195, *p* = 0.003).

The EG showed two positive correlations between level of consciousness (LCF) and control trunk (TCT) rho = 0.81, as well as global functioning (FIM) and trunk control (TCT) rho = 0.96. In the CG, we found similar correlations between global functioning (FIM) and trunk control (TCT) rho = 0.76, as well as level of consciousness (LCF) and trunk control (TCT) rho = 0.88. 

Caregivers in the EG were also interviewed about their level of satisfaction with the training of their loved ones. About 90% of them were satisfied with the medical and hospital staff, and with rehabilitation intervention. Also, after all training sessions, 100% of the caregivers would recommend the treatment in the future.

## 4. Discussion

As far as we know, this is the first study evaluating the clinical and neurophysiological effects of a protocol of multi-sensory and emotional stimulation provided by Neurowave in MCS patients compared to a conventional SS treatment.

### 4.1. Clinical Effects of Neurowave Multi-Sensory and Emotional Stimulation 

Our results showed clinical improvements in consciousness state (LCF, and SECONDs), trunk control (TCT), global functioning (FIM) and communication functions (GC) in our sample of patients with MCS, after our innovative protocol of multi-sensory and emotional stimulation through the Neurowave. Clinical improvements were also achieved by CG, but to a lesser extent than EG. 

Multi-sensory stimulation is a simple and potentially effective approach, which can include auditory (reading/speaking to the patient), visual (i.e., by showing images or photos), olfactory (i.e., presenting his/her favorite smells), and tactile (i.e., touching different materials) stimuli [42]. Our results are in line with previous findings that have already demonstrated the efficacy of multi-sensory stimulation programs. For example, Pape et al. [43] noticed significant gains in arousal and awareness in people with DoC after familiar auditory sensory training. However, according to Norwood et al. [42], MCS patients need at least four sensory stimulation targets [42]. This great involvement is based on the “whole of brain” approach: synchronized communication across several brain regions is needed to maintain consciousness. Indeed, personalized approaches may result in more intense training with a stronger emotional content that may encourage stronger cortical responses. In addition, the stimuli provided should be familiar for the patients [44]. 

Moreover, our experimental training with the Neurowave provided different stimuli, which were also meaningful for the patients in a repetitive and task-oriented manner. Neutral stimuli might be useful to compare patients’ data but can be associated with a high number of false negatives, because they cannot personally engage patients [45]. In line with this hypothesis, it has been shown that personalized stimuli enhance the probability of eliciting a cerebral response in patients affected by DoC [46,47]. 

In our study, the implementation of a multisensory and emotional protocol using the Neurowave has led to better results than conventional training, especially in global communication. We are not completely able to state the reason why this may have occurred, but it is conceivable that the use of the innovative tool was able to furnish more intensive, repetitive and task-oriented stimulation with a consequent boosting of neuroplasticity and functional recovery. Non-verbal communication mainly depends on the right brain hemisphere, and it involves somato-sensory, especially the visual one, and emotional systems. Rasmus et al. [48] found that MCS patients communicate with the use of preverbal level, including the sensory and the behavioral organization level. For these reasons, the Neurowave, which is an emotional multi-sensory approach, might have facilitated an increase in the intra-psychic communication level. Improvements in global communications skills in MCS patients are essential in nursing care and to interact with patients’ caregivers. The relationship between emotional aspects and communication skills is known and it could be relevant in the rehabilitation processes [49,50,51]. For instance, as MCS patients show reduced motor abilities, this may decrease social interactions with worse outcomes for both patients and caregivers [52]. This is why we consider our results clinically significative since our MCS patients, which showed improved global communication, have a better chance to get involved in social interactions, both with medical/nursing staff and their caregivers.

We also found a significative statistical difference between EG and CG regarding level of consciousness (SECONDs). This could be explained by the fact that, as reported by other authors [5,53,54], consciousness recovery depends not only on the type of stimulation (positive, negative vs. neutral stimuli), but also on the possibility to provide such stimulation in an intensive and repetitive way, like the Neurowave can do. In addition, the presence of emotional content (e.g., sight of a familiar face), as proposed in our NES protocol, is known to promote oxytocin release, a hormone that controls key aspects of human behavior [54,55]. The reaction to a familiar emotional content increases the salience of the stimuli, due to its “affective meaning” based on prior experiences, which causes the individual to recreate the experience, even if they are not fully experiencing it. In fact, we hypothesized that all these affective aspects are relevant to support global DoC recovery, particularly to promote the global communication abilities (verbal and not) and awareness in DoC, as we already demonstrated in other papers [27,56,57]. 

This is why, in our opinion, this study has led to potentially important results, since to potentiate communication is fundamental to achieve a better management of these fragile and vulnerable patients for both healthcare professionals and caregivers.

However, the Neurowave is not the only advanced rehabilitation tool in the DoC field, and this is may be why it has not been implemented in clinical practice yet. Despite its higher costs than conventional SS, it is a suitable tool because it can be easily placed next to the bedside and allows both EEG evaluation and personalized treatment. Nonetheless, a cost-effectiveness analysis should be performed in order to investigate whether and to what extent effectiveness is more important than economic sustainability. 

Compared to conventional sensory stimulation, Neurowave offers the advantage to realize a personalized task-oriented assessment and training (emotion—based), optimizing the efforts of the rehabilitation staff, supported by caregiver’s satisfaction, and to allow a simultaneous acquisition of neurophysiological data.

### 4.2. Neurophysiological Effects of Neurowave Emotional Stimulation (NES)

Another important difference between NES and conventional SS is that the tool can also personalize the sensory–emotional training by monitoring the cortical response, using those stimuli that are more meaningful for the patient. The cerebral response following the presentation of the multi-sensory stimulation carried out through this innovative tool has been registered through P300. The P300 is the third positive wave of ERP, and it is considered as the most appropriate cognition-related wave that can evaluate consciousness in DoC [58,59]. In this vein, Li et al. [60] found that P300 can be used as prognostic factor, predicting patients who are more likely to recover. The neurophysiological assessment using the P300 can help the clinicians in either diagnosis/classification of DoC patients or prognosis and management. In this sense, the use of Neurowave in clinical settings could allow a tailored rehabilitation approach for patients with MCS, providing both an objective assessment of brain activity and a concurrent personalized rehabilitation treatment. 

### 4.3. Caregivers’ Anxiety and Satisfaction of the Experimental Method

Our secondary aim was to assess the caregivers’ anxiety and satisfaction about the experimental method we used to stimulate MCS. Specifically, we found reduced levels of caregivers’ anxiety in both two groups, although the caregivers of EG group achieved better scores than CG at SAS. These results might be influenced by the improvements of both motor and cognitive functions obtained by patients [56]. Moreover, each caregiver had a co-therapeutic function, supporting the global recovery of MCS patients as an integral part of the rehabilitation process. In addition, caregivers were supported by the multidisciplinary rehabilitation team during the whole rehabilitation process. This may also explain the improvements in the psychological status of caregivers, since they had an active participation in the care and decision-making processes, as demonstrated by our previous research [57,61]. 

Finally, the EG caregivers, who are the familiars of the MCS patients, achieved high levels of satisfaction about the innovative rehabilitation treatment through Neurowave, likely for the improvements in awareness achieved by MCS patients. 

Our study has some limitations that need to be acknowledged. First, the small sample size does not allow a generalization of our results to the DoC patient population. The retrospective nature of the study is a bias itself and RCT should be fostered to confirm if this innovative tool may lead to better outcomes than conventional treatment. Moreover, we did not investigate the long-term effects of Neurowave, so we are not able to state if and to what extent the improvement in awareness and motor functions lasts. 

## 5. Conclusions

In conclusion, the multi-sensory stimulation approach through Neurowave is an innovative rehabilitation treatment to implement in clinical practice. In fact, after the application of our experimental protocol we found an improvement in cognitive function and global communication, this being fundamental in patients with DoC. Our approach joined the emotional stimuli with auditory and visual feedback, providing the registration of brain activity through the P300. In this way, clinicians could have better information of patients’ levels of motor and cognitive disability, as suggested by our results. This aspect is important to establish a personalized rehabilitation treatment and a correct prognosis, even more in patients with MCS. In a future perspective, further and larger studies should be fostered to confirm these promising findings.

## Figures and Tables

**Table 1:** Social demographic and clinical characteristics of patients and their caregivers at baseline (T0).

	Participants	EG	CG	*p*-Value
**Patients**				
Age (Years)	51.8 (15.32)	50.76 (16.47)	52.95 (14.4)	0.72
Gender				0.24
Male	24 (57.14%)	13 (61.9%)	11 (52.3%)	
Female	18 (42.8%)	8 (38.1%)	10 (47.7%)	
Education (years)				
Elementary school				
Middle schooL	6 (14.28%)	3 (14.3%)	3 (15%)	
High school	20 (47.6%)	11 (52.4%)	9 (45%)	
University	15 (35.7%)	7 (33.3%)	8 (40%)	
	0 (0.00)	0 (0.00)	0 (0.00)	
Aetiology				0.13
Vascular	30 (71.42%)	17 (80.9%)	15 (71.42%)	
Traumatic	11 (26.19%)	4 (19.1%)	6 (28.58%)	
**Caregivers**				
Age	52.8 (11.4)	55.3 (9.53)	50.3 (12.78)	0.2
Gender				0.52
Male	16 (38.09%)	7 (33.3%)	9 (42.9%)	
Female	26 (61.90%)	14 (66.7%)	12 (57.1%)	
Education (years)				0.93
Elementary school	9 (21.4%)	4 (19%)	5 (23%)	
Middle school	15 (35.7%)	8 (38.1%)	7 (33.3%)	
High school	11 (26.1%)	6 (28.6%)	5 (23.8%)	
University	7 (16.6%)	3 (14.3%)	4 (19%)	
Degree of kinship				0.78
Mother/Father	12 (28.5%)	6 (28.6%)	6 (28.6%)	
Husband	12 (28.5%)	6 (28.6%)	6 (28.6%)	
Wife	11 (26.19%)	6 (28.6%)	5 (23.8%)	
Son	2 (4.76)	1 (4.8%)	1 (4.8%)	
Daughter	3 (7.14%)	2 (9.5%)	1 (4.8%)	
Nephew	2 (4.76%)	0 (0.00)	2 (9.5%)	

Legend: EG (Experimental group), CG (Control group).

**Table 3:** Statistical comparison of the clinical score variations from baseline to post-treatment between the experimental group (Neurowave stimulation) and control group (conventional multisensory stimulation); scores are in median (first–third quartiles).

	Outcome Measure	Median (First–Third Quartile) at T0–T1	*p*-Value (T0–T1)
EG	LCF	3(2–5)–3 (2–7)	**0.005**
	SECONDs	3(2–5)–6 (3.5–7)	**0.0002**
	FIM	18 (18–24)–18 (18–30)	**0.004**
	TCT	0(0–12)–0 (0–14.5)	**0.007**
	GC	16(15–17)–20 (16–21.5)	**<0.001**
	SAS	55(47–65)–44 (38–54)	**<0.001**
	**P300 in m/s**	396(387–420.5)–380(358.5–390)	**<0.001**
CG	LCF	3 (1–6.5)–4 (2–7)	**0.01**
	SECONDs	3 (2–6)–5 (2–6)	**0.006**
	FIM	20(18–30)–20 (18–33)	0.13
	TCT	0(0–30.5)–0 (0–40)	0.17
	GC	16(13–17)–17 (14–19.5)	**0.02**
	SAS	50(45–60)-45 (39.5–57)	**0.001**

Legend: EG (Experimental group), CG (Control group), LCF (Level of cognitive functioning), SECONDs (Simplified Evaluation of Consciousness Disorders), TCT (Trunk control test), FIM (Functional independence measure), GC (Global communication), SAS (Zung Self-Rating Anxiety Scale). Statistical significances are in bold.

**Table 4:** ANCOVA results for each covariance model.

Clinical Assessment	Group Coefficient				Adjusted R^2^
	Estimate	Std. Error	t Value	*p* Value	
**LCF**	0.241	0.239	1.004	0.322	0.81
**SECONDs**	0.643	0.175	3.685	**<0.001**	0.86
**TCT**	0.461	1.139	0.405	0.688	0.94
**FIM**	−8.628	4.295	2.009	0.052	0.72
**GC**	1.126	0.420	2.682	**0.011**	0.67
**SAS**	8.457	4.305	1.964	0.057	0.87

Legend: LCF (Level of cognitive functioning), SECONDs (Simplified Evaluation of CONsciousness Disorders), TCT (Trunk control test), FIM (Functional independence measure), GC (Global communication), SAS (Zung Self-Rating Anxiety Scale). Statistical significances are in bold.

## Data Availability

The data presented in this study are available on request from the corresponding author. The data are not publicly available due to the privacy of research participants.
